# Aloin promotes cell apoptosis by targeting HMGB1-TLR4-ERK axis in human melanoma cells

**Published:** 2020-05-14

**Authors:** Pan Li, Kai Ren, Yin yin Liang, Ji kai Liu, Zhuo wen Liang, Yong feng Zhang

**Affiliations:** 1 Medical Research Institute, Northwestern Polytechnical University, Xi′an, China; 2 Institute of Orthopedic Surgery, Xijing Hospital, Fourth Military Medical University, Xi'an, China; 3 Department of Cardiovascular Surgery, Xijing Hospital, Fourth Military Medical University, Xi'an, China

**Keywords:** Aloin, melanoma, apoptosis, HMGB1, ERK signal pathway

## Abstract

Aloin (ALO) is the major anthraquinone glycoside purified from the Aloe species. It is well known for its anti-tumor effects. However, the protective effects of ALO in melanoma cancer and underlying molecular mechanism remain unclear. High-mobility group protein B1 (HMGB1) is an intracellular protein, which has closely association with cell survival, proliferation and metastasis in various cancers. In this study, we explored the effect of ALO on cell survival and apoptosis by targeting HMGB1 signal pathway. We confirmed that ALO exerts a strong effect on promoting cell apoptosis of melanoma cells *in vitro*. Furthermore, HMGB1 release was significantly inhibited in melanoma cancer cells treated with ALO. Knockdown of HMGB1 could enhance melanoma cell death that is induced by ALO treatment. Moreover, HMGB1 facilitated ALO mediated melanoma cell apoptosis by binding to its receptor, Toll-like receptor 4 and activating extracellular regulated protein kinases (ERK) signal pathway. Altogether, our study demonstrated that ALO plays an important role in promoting apoptosis of melanoma cells by inhibiting HMGB1 release and activation of downstream ERK signal pathway.

## Introduction

Melanoma is one of the most common malignant tumors, with a high incidence and poor prognosis (Hopkins et al., 2019[5]). Current treatments include various surgical and immunotherapeutic approaches, such as checkpoint inhibitors. But due to the high cost and complicated individualized genetic background, the therapeutic efficacy is not satisfactory (Mancini et al., 2019[10]). Therefore, it is imperative to seek more effective strategies for treatment of melanoma.

Aloin (ALO) is a major anthraquinone glycoside purified from the Aloe species. It has been used in Chinese medicine to treat or prevent disease for a long term (Park et al., 2009[13]). Many studies have demonstrated that ALO has anti-tumor (Pan et al., 2013[11]), anti-inflammatory (Park et al., 2011[12]), anti-oxidative and immunomodulatory activities (Lee et al., 2019[7]). Its effect on induction of apoptosis has been extensively investigated, especially for cancer cells. Previous studies have reported that ALO facilitates cell apoptosis in various cancers, such as lung (Wan et al., 2017[17]), colorectal carcinoma (Pan et al., 2013[11]), breast cancer (Esmat et al., 2006[4]) and gastric cancer cells (Tao et al., 2019[16]). In melanoma, ALO was demonstrated to enhance cisplatin antineoplastic activity (Tabolacci et al., 2013[15]), but whether ALO could induce melanoma cell apoptosis is still unknown. 

High Mobility Group Box 1 (HMGB1) is a highly conserved nuclear DNA-binding protein that contributes to the stability of genome in physiological conditions. HMGB1 can also be released into extracellular space by active secretion or passive release. Extracellular HMGB1 acts as the “secondary biological effects” by binding with its receptors, such as receptor for advanced glycation end products (RAGE) or Toll-like receptors (TLRs), then activates downstream signal pathways (Cui et al., 2019[2]). HMGB1 is highly expressed in many malignant tumors and reportedly plays an important role in tumor initiation, proliferation and metastasis (Dyer and Rosenberg, 2015[3]; Lee et al., 2019[8]). It is also considered as an early biological target of many malignant tumors. Inhibition of HMGB1 enhances hepatoma cell apoptosis induced by doxorubicin (Lu et al., 2018[9]). In addition, the correlation of HMGB1 and melanoma has been reported. Previous studies demonstrated that UV induced release of HMGB1 inhibited melanocytes apoptosis, and knockdown of HMGB1 enhanced cell apoptosis of melanoma cell (Zhang et al., 2019[20]). Altogether, these studies suggested that HMGB1 is a critical protein and may be considered as a novel therapeutic target in melanoma.

Thus, the present study investigated the effect of ALO on induction of melanoma cell apoptosis and the underlying mechanism. We suggested that ALO induced melanoma cell apoptosis by inhibiting release of HMGB1 release and activation of ERK signal pathway.

## Materials and Methods

### Reagents

ALO and recombinant human HMGB1 were purchased from Sigma Chemical Co. (St. Louis, MO), ERK signal pathway inhibitor, U1026 was purchased from Abcam (Cambridge, MA), specific antibody against HMGB1 (Product No. 6893S), RAGE (Product No. 6996S), TLR2 (Product No. 2229S), LaminA (Product No.4777 ) were purchased from Cell Signaling Technology (Danvers, MA), specific antibody against ERK1/2 (Product No. Ab54230), phosphorylated-ERK1/2 (Product No. Ab207470), PARP (Product No. Ab32138), cleaved-PARP (ab32561), BCL-2 (Product No. Ab32124), Caspase-3 (Product No. Ab184787), Cleaved caspase-3 (Product No. Ab2302), TLR4 (Product No. ab13867) and β-actin (Product No. Ab8226) were purchased from Abcam (Cambridge, MA). HMGB1 ELISA kit (Product No. 326056538) was purchased from Shino-test Corporation (Japan). Annexin V/PI apoptotic kit (KGAV116) was purchased from KeyGen Biotech Co., Ltd. (Nanjing, China). BCA protein assay kit (Product No. P 0010) and CCK8 kit (Product No. C0037) were purchased from Beyotime Institute of Biotechnology (Haimen, China). 

### Cell culture and treatments

Human melanoma cell line, A375 were obtained from GuangZhou Cellcook Biotech Co., Ltd. (Guangzhou, China). Cells were cultured in DMEM medium supplemented with 10 % fetal bovine serum (Thermo Fisher Scientific, Waltham, MA, USA) and 1 % penicillin-streptomycin (Thermo Fisher Scientific) in cell incubator at 37 °C. Cells were treated with different doses of ALO (25, 50, 100 and 200 μg/ml) for 24 h. U1026 was used at a concentration of 20 μM. rhHMGB1 was used at concentration of 100 ng/ml.

### Cell transfection

To regulate the expression of HMGB1 in cells, exogenous plasmid expressing HMGB1 or small interfering RNA (siRNA) were applied as indicated. For transfection, cells were seeded in 6-well plate at density of 3×10^5^ per well for 24 hours, then transfection was carried out by using lipofectamine 3000 reagent (Invitrogen, USA) as recommended by the manufacturers. HMGB1 gene was inserted into pCMV plasmid (pCMV - HMGB1) for mammalian cell expression (GenePharma, Shanghai, China). The siRNA for HMGB1 were commercially available purchased from GenePharma company. The sequences are as follows: Ctrl siRNA sense: TGCATAGGAGTTGGAGAGGTT, antisense: CCTCTCCAACTCCTATGCATT. siHMGB1-1 sense: AGAUAGUUUUCAUCCAUAATT, antisense: UUAUGGAUGAAAACUAUCUCA. siHMGB1-2 sense: CUUUCAUAUAGUUAGCUAATT, antisense: UUAGCUAACUAUAUGAAAGGA. siHMGB1-3 sense: GGAUUAUUAGAAUCAAACATT, antisense: UGUUUGAUUCUAAUAAUCCCA.

### Cell viability assay

Cells were cultured in a 96-well plate at density of 5000 per well. Cell viability was detected by CCK8 assay according to the manufacturer’s instructions.

### Apoptosis assay

Cells were seeded in 6-well plates at density of 3×10^5^ per well for 24 hours. Then cells were harvested with trypsin for further analysis. To analyze apoptosis, the cells were washed in binding buffer and stained with FITC and annexin V conjugated with PI according to manufacturer’s instructions in apoptosis detection kit, and assessed by flow cytometer to determine the apoptosis rate of cells. (The apoptosis rate includes early apoptosis and late apoptosis).

### RNA isolation and qRT-PCR analysis

Total RNA was extracted from cells by using Trizol reagent (Invitrogen), then RNA was reversely transcribed to cDNA by using PrimeScript RT reagent Kit (Takara, Ohtsu, Japan). qRT-PCR analysis was performed using SYBR Premix Ex Taq II (TaKaRa) with the iQ5 PCR Detection System (Bio-Rad, Hercules, CA). The relative mRNA expression was normalized to the β-actin gene. The primers used in this study were as follows: HMGB1 forward: AAACCGATAGGAAACGAGGC, reverse: TCGTGCACCGAAAGTTTCAA. β-actin forward: ACAGAGCCTCGCCTTTGC, reverse: GCGGCGATATCATCATCC.

### Western blotting

The cells were harvested rapidly by sedimentation and nuclear and cytoplasmic extracts were prepared on ice. Each protein sample was quantified using BCA protein assay kit (beyotime, Shanghai, China). Proteins were separated by sodium dodecyl sulfate-polyacrylamide gel electrophoresis and blotted onto a nitrocellulose membrane, then the membranes were blocked in tris-buffered saline solution containing 5 % non-fat dry milk for 2 h, followed by incubating with primary antibody at 4 °C overnight. After washing, membranes were incubated with HRP-conjugated secondary antibody for 2 h at room temperature. β-actin or laminA was used as a loading control for cytoplasmic or nuclear extracts, respectively. 

### ELISA assay

HMGB1 released from A375 cells was detected by using ELISA kit (Shino-test Corporation, Japan) according to manufacturer’s instruction. 

### Statistical analysis

All experiments were repeated three times, the results are described as the mean ± SD of three different determinations. Western blotting bands were quantified by using ImageJ software. Data analysis was performed using GraphPad Prism version 6.0 software (GraphPad Software, San Diego, CA), Data were analyzed by Student’s t test and differences were considered significant when p<0.05.

## Results

### Aloin reduced A375 cell viability and induced cell apoptosis

**Figure 1:**
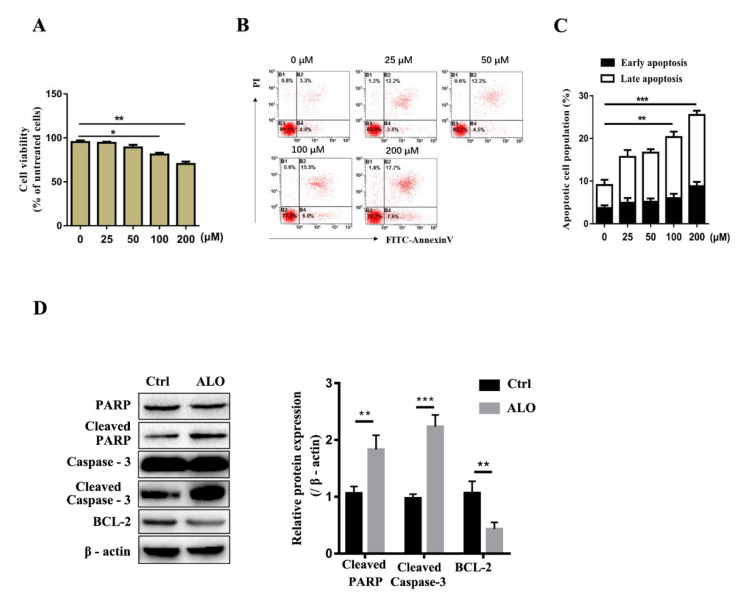
ALO reduced A375 cell viability and induced cell apoptosis. (A) A375 cell viability was measured by CCK8 assay. (B) A375 cell apoptosis was detected by flow cytometry. (C) The statistical data for apoptosis detection. (D) The apoptotic-related protein expression was detected by Western blotting. The blots shown are representative blots of three replicates. *p < 0.05, **p < 0.01, ***p < 0.001.

To detect the effect of ALO on A375 cells we first analyzed the cytotoxic effect of ALO on A375 cells. The CCK8 assays showed that 100 and 200 μM ALO could significantly reduce the cell viability of A375 (Figure 1A(Fig. 1)). Subsequently, we tested the effect of ALO on A375 cell apoptosis by flow cytometry through signaling of Annexin V and PI staining, the results showed that cell apoptosis rate was significantly increased in both 100 and 200 μM ALO treated group (early apoptosis: 6.0 %, 7.8 %, late apoptosis: 15.5 %, 17.7 %, respectively) compared with control group (early apoptosis: 4.8 %, late apoptosis: 3.3 %) (Figure 1B and C(Fig. 1)), indicating that ALO could significantly promote cell apoptosis of A375. Furthermore, we analyzed apoptotic-related protein expression in A375 cells after treatment with 100 μM ALO, Western blotting results showed that ALO enhanced expression of pro-apoptotic proteins, cleaved-PARP and cleaved-caspase-3, but reduced the expression of anti-apoptotic protein, such as BCL-2 (Figure 1D(Fig. 1)). Altogether, these results demonstrated that ALO exerts cytotoxicity on A375 cells by reducing cell viability and promoting cell apoptosis. 

### ALO inhibits the expression and release of HMGB1 from A375 cells

**Figure 2:**
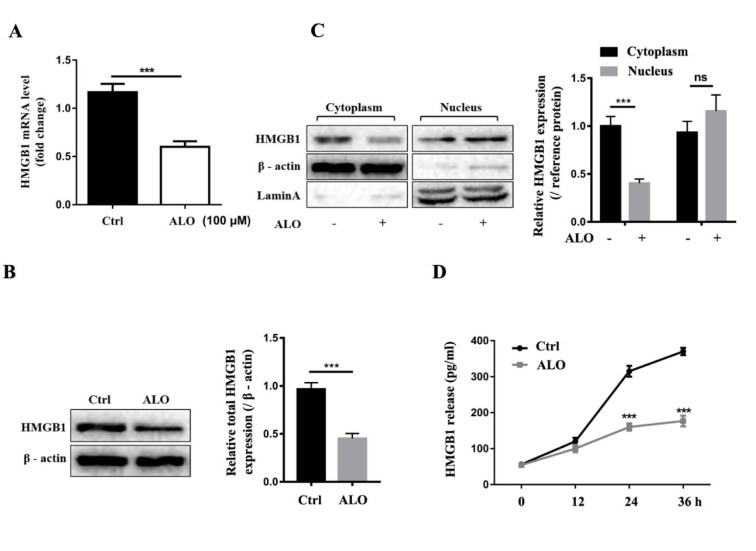
ALO inhibits the expression and release of HMGB1 from A375 cells. (A) HMGB1 mRNA level was detected by qRT–PCR assay. The expression of (B) total HMGB1 and (C) cytoplasm and nucleus HMGB1 level in A375 cells was detected by Western blotting (β- actin and laminA were used as reference protein for cytoplasm HMGB1 and nucleus HMGB1, respectively). (D) The release of HMGB1 in A375 cells after ALO treatment for different time was detected by Elisa assay. The blots shown are representative blots of three replicates. ***p < 0.001.

Previous studies found that HMGB1 is highly expressed in human melanoma tissue and suggested it as a potential therapeutic target for melanoma treatment. Thus, we examined whether ALO could reduce HMGB1 expression in A375 cells. To testify our hypothesis, we treated A375 cells with ALO at concentration of 100 μM for 24 hour *in vitro*, qRT-PCR analysis showed that HMGB1 mRNA expression was inhibited in ALO treatment group (Figure 2A(Fig. 2)), correspondingly, Western blotting results also showed that total HMGB1 expression was decreased (Figure 2B(Fig. 2)). Given that HMGB1 is located in nuclear physiologically, but is transferred into cytoplasm and then released into extracellular under pathological condition, we further tested whether ALO could inhibit translocation of HMGB1. By isolating proteins from cytoplasmic and nuclear, we found that ALO dramatically decreased the cytoplasmic HMGB1 level compared with control group, indicating that ALO inhibited the translocation and release of HMGB1 (Figure 2C(Fig. 2)). Subsequent ELISA assay also demonstrated that ALO could reduce the accumulation of HMGB1 in supernatants of A375 cells (Figure 2D(Fig. 2)). Our findings verified that ALO can reduce HMGB1 expression and release in A375 cells.

### HMGB1 affects ALO-induced A375 cell apoptosis

**Figure 3:**
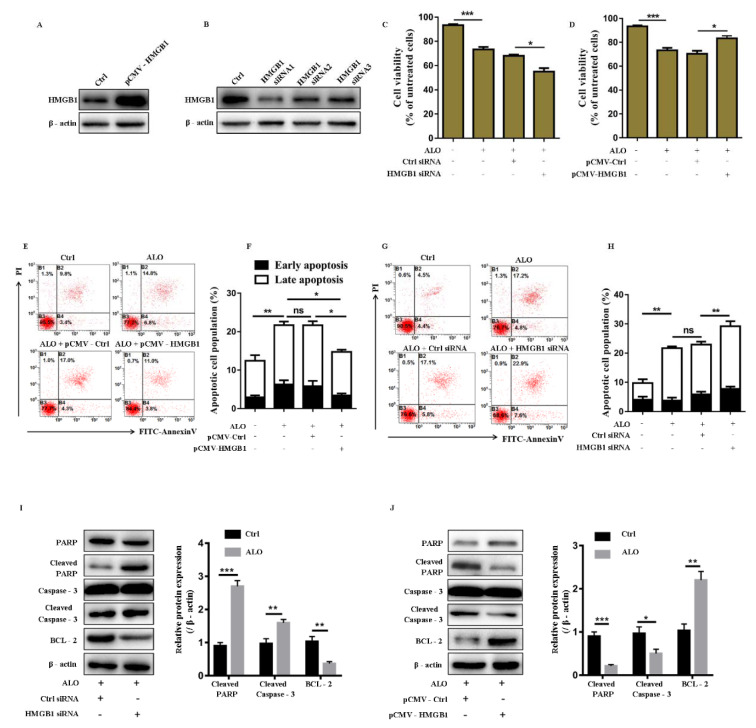
HMGB1 affects ALO-induced A375 cell death. (A) The transfection efficiency of pCMV - HMGB1 was detected by Western blotting. (B) The transfection efficiency of HMGB1 siRNA was detected by Western blotting. (C) The cell viability of A375 transfected with control siRNA or HMGB1 siRNA and (D) pCMV - Ctrl or pCMV - HMGB1 before ALO treatment was detected by CCK8 assay. (E and G) The representative charts for flow cytometry show cell apoptosis rate in different groups. (F and H) The statistical data for cell apoptosis rate of A375 transfected with pCMV - Ctrl or pCMV - HMGB1 and control siRNA or HMGB1 siRNA before ALO treatment. (I) The apoptotic-related protein expression was detected in A375 cells transfected with control siRNA or HMGB1 siRNA and (J) pCMV - Ctrl or pCMV - HMGB1 by Western blotting. The blots shown are representative blots of three replicates. *p < 0.05, **p < 0.01, ***p < 0.001.

To test whether HMGB1 was involved in ALO induced A375 cell apoptosis, we regulated HMGB1 expression in A375 cells by transfection with exogenous plasmid expressing HMGB1 (pCMV- HMGB1) or siRNA for HMGB1 (HMGB1 siRNA) *in vitro*. The transfection efficiency was firstly confirmed by Western blot (Figure 3A and B(Fig. 3)). Then, we compared the cell viability and apoptosis rate of A375 cells under condition of HMGB1 overexpression or HMGB1 knockdown before ALO treatment. CCK8 assay showed that inhibition of HMGB1 further increased the cytotoxic effect of ALO on A375 cells, while overexpression of HMGB1 can reduce the cytotoxic effect of ALO (Figure 3C and 3D(Fig. 3)). 

Flow cytometry analysis also demonstrated that enhanced HMGB1 expression could reduce ALO-induced cell apoptosis (Figure 3E and F(Fig. 3)), while inhibition of HMGB1 increased cell apoptosis in A375 cells (Figure 3G and H(Fig. 3)). Moreover, we also detected the expression of apoptotic-related proteins, Western blotting assays showed that HMGB1 inhibition further increased expression of cleaved-PARP and cleaved-caspase-3, and decreased the expression of BCL–2 (Figure 3I(Fig. 3)), and vice versa (Figure 3J(Fig. 3)), indicating that HMGB1 inhibition promoted A375 cell apoptosis induced by ALO. These findings suggested that HMGB1 was involved in ALO-induced A375 cell apoptosis.

### ALO inhibits HMGB1-induced TLR4-ERK signal pathway activation

**Figure 4:**
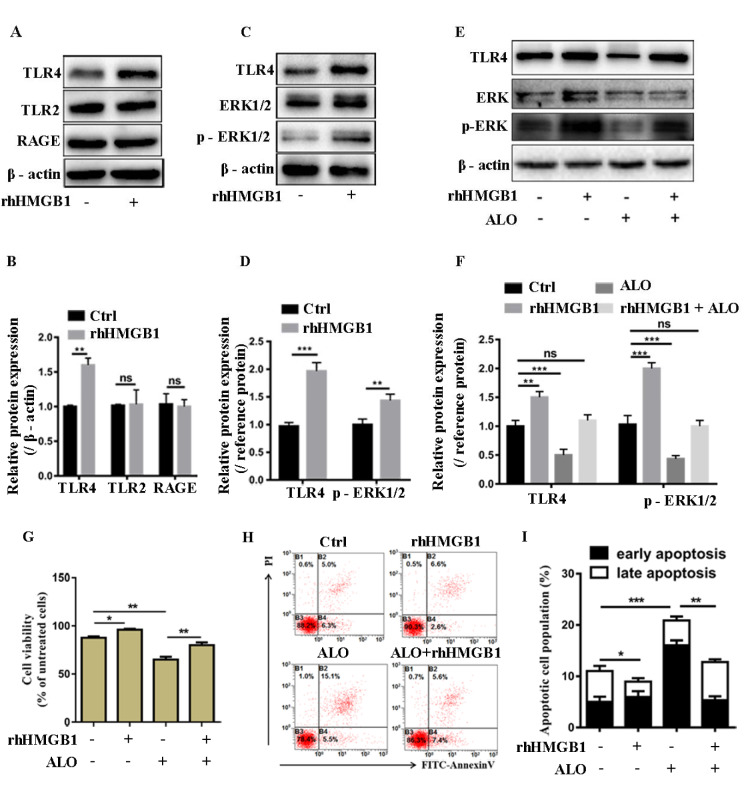
ALO inhibits HMGB1 induced TRL4-ERK signal pathway activation. (A and B) The receptors for HMGB1 in A375 cells after treatment with rhHMGB1 were detected by western blotting. (C and D) The phosphorylated ERK in A375 cells after treatment with rhHMGB1 were detected by Western blotting (β-actin and total ERK1/2 were used as reference protein for TLR4 and p-ERK, respectively). (E and F) The receptor for HMGB1, TLR4 and the phosphorylation level of ERK in A375 cells treated with rhHMGB1 only or combined with ALO were detected by Western blotting (β-actin and total ERK1/2 were used as reference protein for TLR4 and p-ERK, respectively). The blots shown are representative blots of three replicates. (G) The cell viability of A375 cells treated with rhHMGB1 only or rhHMGB1 combined with ALO were detected by CCK8 assay. (H and I) The cell apoptosis rate of A375 treated with rhHMGB1 only or combined with ALO was detected by flow cytometry. The concentration for rhHMGB1 was 100 ng/ml. **p < 0.01.

Previous studies have demonstrated that HMGB1 promotes melanoma cell survival through activating the downstream ERK signal pathway, in this study, we further tested the effect of ALO on this signal pathway. Given that RAGE, TLR2 and TLR4 are three main receptors for HMGB1, we first determined whether these receptors were expressed in A375 cells, we treated cells with recombinant human HMGB1 (rhHMGB1) at concentration of 100 ng/ml for 48 hours as described in previous studies. We found that all of the three receptors were expressed in A375 cells, especially, rhHMGB1 stimulation can significantly increase the expression of TLR4, but not RAGE or TLR2 (Figure 4A and B(Fig. 4)), indicating TLR4 may be the more important one in HMGB1 mediated A375 cell survival. Then we verified that rhHMGB1 stimulation can activate ERK signal pathway by promoting the phosphorylation level of ERK in A375 cells (Figure 4C and D(Fig. 4)). Subsequently, we analyzed the effect of ALO on the expression of TLR4 and the phosphorylation of ERK. As we can see in Figure 4E and F(Fig. 4), ALO significantly inhibited the expression of TLR4 and the phosphorylation level of ERK, but exogenous addition of rhHMGB1 can reverse this process. Correspondingly, rhHMGB1 treatment could increase the cell viability (Figure 4G(Fig. 4)) and rescue cell apoptosis induced by ALO in A375 cells (Figure 4H and I(Fig. 4)). These results indicated that ALO could inhibit the activation of ERK signal pathway induced by HMGB1 in A375 cells.

### Blocking ERK signal pathway enhanced ALO-induced A375 cell apoptosis

**Figure 5:**
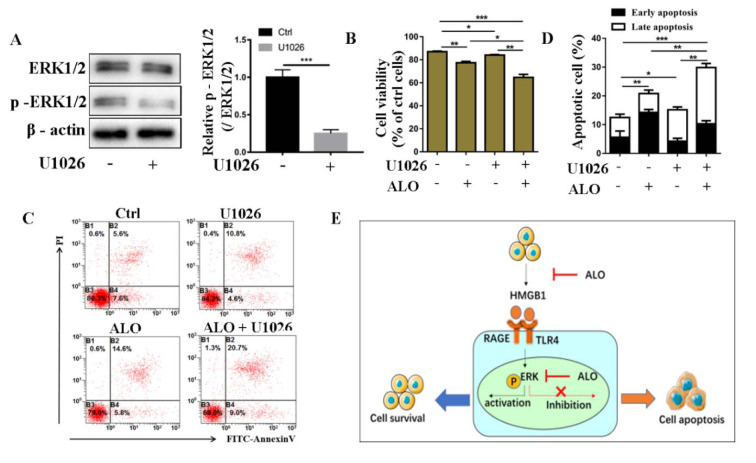
Inhibition of TRL4-ERK signal pathway enhanced ALO-induced A375 cell apoptosis. (A) The ERK and phosphorylated ERK in A375 cells stimulated with U1026 at concentration of 20 μM were detected by Western blotting. The blots shown are representative blots of three replicates. (B) The cell viability of A375 treated with ALO combined with U1026 was detected by CCK8 assay. (C) The representative charts for flow cytometry show cell apoptosis rate in different groups. (D) The statistical data for cell apoptosis rate of A375 treated with ALO combined with U1026. (E) The diagrammatic drawing for this study. **p < 0.01, ***p < 0.001.

To further test the effect of ERK signal in ALO-induced A375 apoptosis, we treated A375 cells with specific inhibitor for ERK signal pathway, U1026 at concentration of 20 μM. The inhibition efficiency was confirmed by Western blotting assays (Figure 5A(Fig. 5)). The results showed that cell variability was decreased in A375 cells treated with ALO combined with U1026 compared with that treated with ALO only (Figure 5B(Fig. 5)), meanwhile, the apoptosis rate was more significantly increased in cells treated with ALO combined with U1026 compared with ALO only (Figure 5C and D(Fig. 5)), suggesting that inhibition of ERK signal pathway could increase the cytotoxic effect of ALO on A375 cells and enhance ALO-induced cell apoptosis. Taken together, our results demonstrated that ALO induced A375 cell death by inhibiting HMGB1-induced ERK signal pathway activation (Figure 5E(Fig. 5)).

## Discussion

The aim of the present study was to evaluate the potential effects of ALO, an active compound isolated from Aloe species, on melanoma cell survival and death. Based on A375 melanoma cell line, our data demonstrated that ALO could inhibit melanoma cell survival and promote cell apoptosis. Furthermore, ALO reduced the expression and release of HMGB1 from A375 cells. Additionally, ALO treatment reduced HMGB1-induced ERK signal pathway activation. These ameliorative effects were similar with the effect of ALO on other cancer cells, such as colorectal carcinoma. Therefore, the present study clarified that ALO has beneficial effects to prevent melanoma cell survival *in vitro*, the *in vivo* studies are required in the future. 

ALO is a traditional Chinese medicine that has been used for a long term in china. Previous studies have been clarified that ALO has strong anti-cancer activities. Wang et al. reported that ALO treatment can induce apoptosis of gastric cancer cells and colorectal cells (Wang et al., 2018[18]). Besides, *in vivo*, ALO is also efficient to ameliorate disease progression in sepsis (Wang et al., 2018[18]) and colorectal mouse model (Pan et al., 2013[11]). In addition, previous study has focused on the effect of ALO on melanoma, and demonstrated that ALO can enhance cisplatin antineoplastic activity in B16-F10 melanoma cells by transglutaminase-induced differentiation (Tabolacci et al., 2013[15]). In this study, consistently, our results also suggested that ALO is effective in ameliorating melanoma disease progression by promoting cell apoptosis. But the limitation is that we did not clarify the effect of ALO on melanoma cell metastasis.

In this study, we also focused on the concentration of ALO on melanoma cell survival and apoptosis. Our results confirmed that ALO inhibited melanoma cell line A375 variability in a concentration-dependent manner, and ALO at concentration of 100 µM is capable of inducing A375 cell apoptosis. Our results firstly identified the effective concentration of ALO in inducing melanoma cell death.

Previous studies have reported that HMGB1 is associated with melanoma initiation and progression, further suggested HMGB1 as a potential therapeutic target for melanoma therapy. Lee et al. demonstrated that HMGB1 depletion causes a senescence-apoptosis shift in B16-F10 melanoma cells (Lee et al., 2019[6]), consistently, our study indicated that down-regulation of HMGB1 expression promotes ALO-induced A375 cell apoptosis, whereas up-regulation of HMGB1 expression inhibits ALO-induced A375 cell apoptosis. Besides, we also find that all of the three receptors, RAGE, TLR2 and TLR4 were expressed in A375 cells under stimulation of rhHMGB1, especially for TLR4, which was highly expressed. These results are in agreement with other studies showing that TLR4 is highly expressed in human melanoma tumors as well as a negative association between the TLR4 expression and relapse free survival (Rossi et al., 2015[14]; Chen et al., 2018[1]; Wei et al., 2019[19]). Activation of ERK signal pathway has been demonstrated to be associated with cell survival, in this study, we also found that ALO induced A375 cell apoptosis by inhibiting activation of ERK signal pathway. 

In conclusion, our results demonstrated that ALO has anti-tumor effects on melanoma cells. Although ALO would not eradicate melanoma, the present study suggests that this kind of approach may be combined with the more conventional cytotoxic chemotherapy or any other methods to interfere with cancer progression. The limitation is no *in vivo* data included in our study. Future, hopefully, will evaluate the results clinically in participants.

## Notes

Pan Li and Kai Ren contributed equally as first authors to this work.

Zhuo wen Liang and Yong feng Zhang (MD PhD, Institute of Orthopedic Surgery, Xijing Hospital, Fourth Military Medical University, 127 West Changle Road, Xi’an 710032, China; E-mail: 349332809@qq.com) contributed equally as corresponding authors.

## Funding

The present study was supported by a grant from National Natural Scientific Foundation of China (grant no. 81572151 and 51907197).

## Availability of data and materials

The datasets used and/or analyzed during the current study are available from the corresponding author on reasonable request.

## Authors' contributions

YF and ZL designed the study. PL, YL made substantial contributions to conception and design, acquisition of data, analysis and interpretation of data and figures. PL and JL wrote the manuscript. TL revised the manuscript critically and advised revisions. All authors have read and approved the manuscript, and take public responsibility for appropriate portions of the content.

## Ethics approval and consent to participate

Not applicable.

## Patient consent for publication

Not applicable.

## Competing interests

The authors declare that they have no competing interests.
